# Water temperature and chlorophyll a density drive the genetic and epigenetic variation of *Vallisneria natans* across a subtropical freshwater lake

**DOI:** 10.1002/ece3.10434

**Published:** 2023-08-15

**Authors:** Yixian Li, Manli Xia, Xuyao Zhao, Hongwei Hou

**Affiliations:** ^1^ The State Key Laboratory of Freshwater Ecology and Biotechnology, The Key Laboratory of Aquatic Biodiversity and Conservation of Chinese Academy of Sciences Institute of Hydrobiology, Chinese Academy of Sciences Wuhan China; ^2^ University of Chinese Academy of Sciences Beijing China

**Keywords:** chlorophyll a, epigenetic variation, genetic diversity, *V. natans*, water temperature

## Abstract

Plant genetic diversity differs in habitat's oscillations, especially species distributed under heterogeneous environmental conditions. Freshwater ecosystems are vulnerable to biotic and abiotic impacts, which affect the genetic and epigenetic variations in aquatic plants. The extent of environmental heterogeneous attributes can be examined based on genetic and epigenetic variations. Such variations under environmental gradient can provide evidence for understanding the correlations between rapid environmental changes and species evolution. In this study, we performed amplified fragment polymorphism length and methylated‐sensitive amplified polymorphism analysis to depict the genetic and epigenetic variations of *Vallisneria natans* in a subtropical lake. Results showed that this species maintained a relatively high genetic diversity (mean *H*_E_ = 0.320, *I* = 0.474, *PPL* = 85.93%) and epigenetic variation (mean e*H*_E_ = 0.282, e*I* = 0.428, e*PPL* = 83.91%). Water body temperature and chlorophyll a density were positively correlated to the genetic and epigenetic variations. The clonal generates of *V. natans* depicted a relative high methylation level and shew ancestral scenario between the genet and the second clonal generation. These findings revealed that species diversity is unevenly distributed under environmental heterogeneity, even at a fine geographic scale. Environmental characteristics in relation to temperature and chlorophyll a should be considered in the analysis of the genetic and epigenetic variations. Additionally, epigenetic variations between genets and ramets should be considered with caution when applied to analysis of other aquatic species.

## Graphical abstract

We analyzed the genetic diversity and epigenetic variation of *Vallisineria natans* in Liangzi Lake and found temperature and chlorophyll density were correlated to the species' genetic diversity and epigenetic variation.Then, based on the associated environmental factors, we constructed the priority restoration areas.

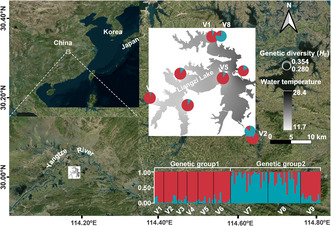

## 1 INTRODUCTION

Macrophyte genetic diversity is often consonant with the adaptability and population sustainability to the living surroundings (Engelhardt et al., 2014). Variations at genetic and epigenetic levels are considered an essential process in repaid adjustments to dynamic habitat conditions for aquatic plants (Schulz et al., 2014; Wang et al., 2020). Knowledge about genetic and epigenetic variations in aquatic plants not only indicates how plants are involved and adapt to different water ecosystems but also provides reasonable suggestions for species management (Crane et al., 2022; Waycott, 1998). Epigenetic variations without changing DNA sequence are regarded as valuable tools for estimating plant adaptation under changing environmental conditions (Verhoeven et al., 2016).

Aquatic ecosystems are vulnerable to biotic and abiotic disturbances (Robertson et al., 2017). In recent years, severe water eutrophication has altered the water body to pollutant source and narrows the suitable habitats for macrophytes (Leitch et al., 2014). Other than habitation oscillation, the adverse effects of food chain disequilibrium have largely exacerbated the maintaining pressure of aquatic plants (Greig et al., 2012). Since the 10 years fishing ban in the Yangtze River basin was implemented in 2020, the propagation pressure of aquatic plants intensifies with the expansion of some herbaceous fish species that prey young sprouts of plants as food. Given these situations, macrophytes have lost their habitats in lakes and ponds, especially in the Yangtze River floodplain of China.

*Vallisneria natans* is a clonal species that proliferates through facultative asexual method; it inhabits in paddy ponds, shallow lakes, and river stretches (Zhou et al., 2016). It is well known for its wide ecological adaptive ability and sensitivity to heavy metals during environmental remediations (Fu et al., 2021). Previous studies reported a high level of genetic diversity (average *H*_E_ = 0.490) in *V. natans* and a relative lower species diversity in *Vallisneria* genera (*H*_E_ = 0.210 in *V. natans* and *H*_E_ = 0.170 in *V. spinulosa*) (Wang et al., 2010). Thus far, epigenetic study of this species and genetic variation analysis within an environmental gradient in natural habitats is lacking.

In this study, we assessed the levels of genetic and epigenetic variations in *V. natans* across Liangzi Lake, which has the highest level of macrophyte diversity in the Yangtze River basin, we also compared the environmental components among habitats. By using amplified fragment length polymorphism (AFLP) and methylated‐sensitive amplified polymorphism (MSAP) markers, we conducted the following: (1) investigated the genetic diversity and epigenetic variations in *V. natans* across the Liangzi Lake; (2) characterized the correlations between environmental factors and genetic/epigenetic variations in *V. natans*; (3) helped build the suitable areas for species restoration.

## 2 MATERIALS AND METHODS

### 2.1 Sampling design and DNA extraction

**FIGURE 1:**
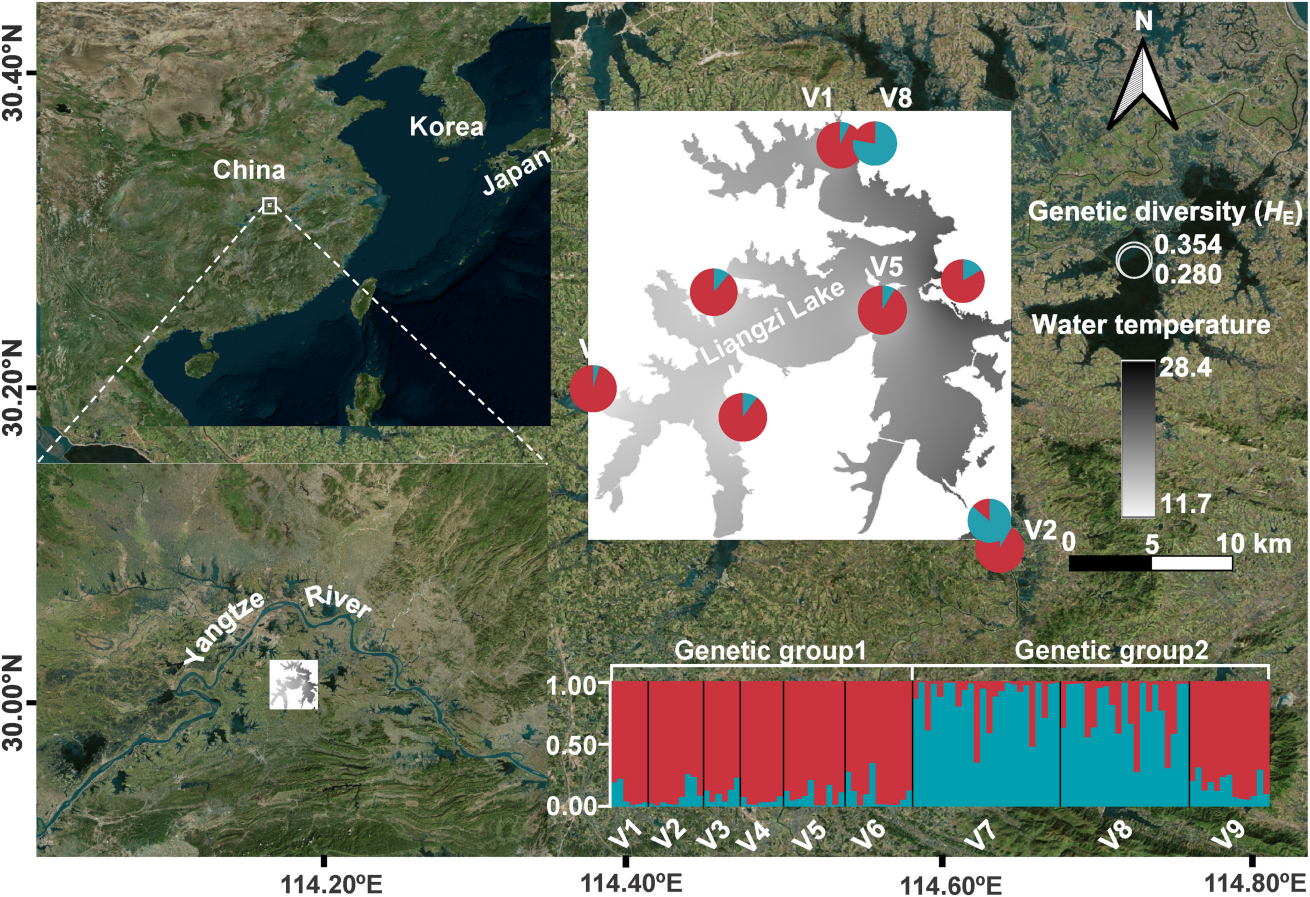
Main characteristics of the study areas: lake location, locations of nine *Vallisneria natans* populations. The assignment of two major amplified fragment polymorphism length genetic clusters among *V. natans* populations is shown in pie charts.

Our study focused *V. natans* populations across Liangzi Lake (Figure 1, Table 1). A total of 109 *V. natans* individuals were collected approximately 10 m from each other at nine sites from January to July in 2020, 22 individuals of the clonal generations and eight individuals of the second clonal generations were also obtained at the same time. The geographic coordinates of the sampling sites were recorded using a global positioning system. Young leaves at the same development stage were collected, immediately dried in silica gel, and stored at −20°C for further use. Total genomic DNA was extracted from approximately 20 mg of dried tissue by using a Plant Genomic DNA Rapid Extraction kit (Tsingke). The integrity of the genomic DNA was checked using 1% agarose gel electrophoresis against a molecular weight standard. DNA concentrations were determined using Nanodrop Spectrophotometer (Thermo Fisher Scientific).

**TABLE 1:** Genetic diversity and epigenetic variation parameters of nine populations of *Vallisneria natans* based on 476 AFLP loci and 392 MSAP loci.

Code	Latitude	Longitude	*N*	Genetic diversity			Epigenetic diversity		
				*H*_E_	*I*	*PPL* (%)	e*H*_E_	e*I*	e*PPL* (%)
V1	30.354	114.535	8	0.321	0.474	84.03	0.306	0.452	80.56
V2	30.098	114.637	9	0.350	0.513	88.66	0.310	0.462	84.64
V3	30.261	114.455	6	0.331	0.485	83.19	0.291	0.431	76.18
V4	30.199	114.379	7	0.326	0.477	81.51	0.289	0.431	78.06
V5	30.249	114.563	10	0.354	0.522	92.02	0.315	0.474	90.60
V6	30.181	114.474	11	0.346	0.510	90.13	0.307	0.463	88.09
V7	30.116	114.631	24	0.281	0.427	86.97	0.214	0.344	83.70
V8	30.355	114.558	21	0.288	0.437	86.97	0.248	0.395	90.60
V9	30.268	114.614	13	0.280	0.419	79.93	0.261	0.402	82.76
Mean				0.320	0.474	85.93	0.282	0.428	83.91

Abbreviations: AFLP, amplified fragment polymorphism length; e*H*_E_, epigenetic Nei's gene diversity; e*I*, epigenetic Shannon's information index; e*PPL* (%), percentage of epigenetic polymorphic loci; *H*_E_, Nei's gene diversity; *I*, Shannon's information index; MSAP, methylated‐sensitive amplified polymorphism; *N*, number of individuals; *PPL* (%), percentage of polymorphic loci.

### 2.2 AFLP and MSAP fingerprinting

Amplified fragment polymorphism length and methylated‐sensitive amplified polymorphism markers were used to evaluate the genetic and epigenetic diversity of *V. natans*. The detailed procedures for AFLP and MSAP analyses and statistical analysis are described in Appendix S1.

An initial selective PCR analysis of eight individuals across four populations was carried out with 12 primer combinations; only the primers that provided clear, reproducible bands with sufficient polymorphic variation between populations were used in the analysis. The eight most informative primer combinations (E‐AGC/M‐CTT, E‐AGC/M‐ CAC, E‐ACA/M‐CTC, E‐ACA/M‐CAT, E‐ACT/M‐CTT, E‐ACT/M‐CGA, E‐ACT/M‐CAA, E‐ACT/M‐CAT) were retained for AFLP analysis. Five primer pairs (E‐ACT/M‐TTA, E‐AGC/M‐TTA, E‐AGC/M‐TTG, E‐ACA/M‐TCT, E‐ACA/M‐TTA) were retained for MSAP analysis. To estimate the error rate in AFLP and MSAP genotyping, we randomly select 12 DNA samples to duplicate the AFLP and MSAP procedure.

### 2.3 Habitats heterogeneity assessment

During the assessment of habitat heterogeneity, 41 environmental variables were analyzed (Table S1). Elevation, pH, water body temperature (WT), Secchi depth (SD), water body conductivity (Cond), total dissolved solids (TDS), salinity (Sal), and dissolved oxygen (DO) were measured in situ with a multiparameter water quality sensor (YSI Company). Total nitrogen (TN), total phosphorus (TP), chemical oxygen demand (COD), and chlorophyll a (*Chl*‐a) were measured using the method recommended by the National Groundwater Analysis Standard Procedure (GB 3838‐2002). The concentrations of six heavy metal elements (As, Cd, Pb, Se, Cu, and Zn) in the water samples were analyzed by inductively coupled plasma‐mass spectrometry (ICP‐MS, NexION2000, PerkinElmer). Distance to the nearest village (DTV), distance to the road (DTR), and number of village populations (NHV) were acquired using QGIS v3.28 (http://www.qgis.org) and through field survey by locals. The global climate and weather database from Worldclim v2.1 (http://worldclim.org) was used to extract 19 bio‐climates attributes at a 30‐arc‐s resolution. Previous studies indicated that multicollinearity in variables might lead to the overfitting of the models, temperature (Bio1‐11) and precipitation (Bio12‐19) were divided into two groups, and Pearson correlation coefficients were calculated to reduce the multicollinearity. One variable in each pair with Pearson's correlation coefficients (*r*) > .8 was eliminated. As topographic slope strongly influences the local temperature and the thermal conditions of the habitat, the slope aspect of each sampling site was analyzed based on the attributes of the topography of Liangzi Lake from the Advanced Spaceborne Thermal Emission and Reflection Radiometer Global digital elevation.

### 2.4 Data analysis

Amplified fragment polymorphism length and MSAP genotypes were obtained from ABI sequencer 3730XL (Thermo Fisher Scientific). Fragments between 100 and 500 bp were detected using GeneMarker v2.2.2 software (Hulce et al., 2011). The polymorphic loci were confirmed manually, and peaks with height less than 20 relative fluorescent units were not scored. The polymorphic binary 0/1 matrix were constructed as presence “1” or absence “0” of the bands. For MSAP datasets, R package msap (Pérez‐Figueroa, 2013) was used to calculate the types of genome methylations present. In MSAP patterns, four patterns were obtained: the presence of both *EcoR*I/*Msp*I and *EcoR*I/*Hpa*II (1/1) indicates unmethylated conditions; fragments present only in *EcoR*I/*Msp*I profiles (0/1) denote cytosine hemi‐methylation; fragments present only in *EcoR*I/*Hpa*II (1/0) are considered as hemi‐methylated CHG sites; and absence of fragments of both profiles (0/0) representing uninformative states caused either by different methylation types or restriction locus polymorphism. To separate the unmethylated and methylated fragments, we divided loci as either methylated‐susceptible loci (MSL) or non‐methylated loci (NMSL) and a 0/1 binary matrix was also constructed where “0” represents a non‐methylated locus and “1” represents a methylated locus.

Indices of genetic diversity and epigenetic variations within populations were obtained from POPGENE v1.3.2 (Yeh, 1999) assuming the Hardy–Weinberg equilibrium, the indices included the following: (1) expected heterozygosity (*H*_E_ and e*H*_E_), (2) mean Shannon's information index (*I* and e*I*), and (3) percentage of polymorphic loci (*PPL* and e*PPL*).

Based on the Jaccard genetic similarity coefficient, an unweight pair group method with arithmetic average (UPGMA) dendrogram was constructed to divide the genetic groups among populations with the R package ape (Paradis & Schliep, 2019). The proportions of genetic variations among groups of populations (ø_CT_), among populations within groups (ø_SC_), and within populations (ø_ST_) were examined by analysis of molecular variance (AMOVA) using the R package poppr (Kamvar et al., 2014). GenAlEx v6.503 (Smouse & Peakall, 2012) was used to characterize genetic and epigenetic differentiation at each locus. The levels of statistical significance were determined after 9999 permutations.

The potential population structure of *V. natans* were inferred from the AFLP dataset by using the Bayesian clustering approach with STRUCTURE v2.3.4 software (Pritchard et al., 2000). The analysis was performed using the admixture ancestry model with correlated allele frequencies; the number of clusters (*K*) was set to vary from 1 to 9, with each run having 5 × 10^6^ Markov chain Monte Carlo (MCMC) iterations following a burn‐in period of 10^6^ steps. The likelihood values were calculated to partition the populations into groups based on different *K* values with STRUCTURE HARVESTER v0.6.94 (Earl & VonHoldt, 2012).

The correlations between genetic diversity, epigenetic variation, geographic distance, and environmental distance were examined. A pair‐wise distance matrix was constructed based on genetic diversity indices (*H*_E_, *I*, *PPL*) and epigenetic indices (e*H*_E_, e*I*, e*PPL*). Bray–Curtis distance was calculated among the samples. The Euclidean geographic distance and Euclidean environmental distance were also computed using the coordinates of sampling sites and environmental variables, respectively. Mantel test was performed using the R package vegan (Oksanen et al., 2007) to detect the relationships between genetic, epigenetic, geographical, and environmental distances with 9999 permutations for significance test.

The importance of environmental variables correlated with genetic diversity and epigenetic variations was accessed by Boruta feature selection “random forest” analysis (*p* < .05) using R package Boruta (Kursa et al., 2020). Random forest (RF) is a robust supervised learning algorithm that can be used for various tasks including evaluating the importance of variance and classification problems. The RF model is commonly used to predict the accuracy of classification when the aim variances are factors, but it can also be used to measure the relative importance of each feature in the prediction. Environmental variables ranked by the RF model in order of importance to genetic and epigenetic variation were determined over 1000 iterations and collected for downstream analysis.

We used Bvstep method in R package sinkr (Taylor, 2017) with a two‐step selection method to select the best combinations of environmental variables correlated with genetic and epigenetic variations. Considering that the linear and nonlinear correlations may exist between genetic/epigenetic variation and environmental variables, we assessed environmental factors associated to genetic/epigenetic indices by performing network analysis using the maximal information coefficient (MIC) scores in R package minerva (Filosi et al., 2012). MIC is an insightful score that reveals positive, negative, and nonlinear associations among variables. The MIC associations were corrected for false discovery rate (FDR < 0.05), and the final networks were constructed with relationships that were found to be statistically significant (*p* < .05) after FDR correction.

Distance‐based redundancy analysis (db‐RDA) was performed using the vegan package to quantify the pure and combined effects of environmental variables on genetic and epigenetic variations. This multivariate ordination technique is used to test whether the variations in one independent variable can explain the variance of another independent variable. Furthermore, the contributions of environmental variables were determined by variance partitioning analysis (VPA) and hierarchical partitioning (HP) using the multiple regression algorithm in the R package rdacca.hp (Lai et al., 2022).

To detect genetic–environmental associations, we used latent factor mixed models (LFMM 2) and detected the correlations between environmental variables and genotypic variations in R package lfmm (Caye et al., 2019). This method is efficient for dealing with false discovery rates, sampling design limitations, and spatially autocorrelated populations. The environmental values associated with genetic and epigenetic variation were scaled and centered for the analysis. We set *K* = 2 and ran 50,000 iterations, with 50% burn‐in and 10 repetitions. Significant genetic and epigenetic loci were retained with thresholds of *p* < .05.

To determine the degrees of environmental contributions at the genetic diversity and epigenetic variation levels, we used the partial least squares path model (PLS‐PM), which can determine complex multivariate relationships among variables, to quantify the direct and indirect effects of environmental variables on genetic and epigenetic variations. The PLS‐PM does not require any distributional assumptions about the data, which are usually difficult to meet in natural ecosystems. We established a model based on the expected relationships and key drivers among environmental variables, genetic variation, and epigenetic variation with the R package plspm (Ravand & Baghaei, 2016). In this model, we compiled variables that were highly linear with respect to water quality as latent variables and performed nonparametric bootstrapping validation (1000 resamples) to estimate the precision of the parameters. A bootstrap confidence interval of 95% was used to judge whether estimated path coefficients were significant. The final model was chosen among all constructed models based on the goodness of fit (GoF > 0.7).

The intrinsic adaptions abilities and epigenetic mutations within genets and ramets were analysis using self‐organizing map (SOM) clustering method in R package kohonon (Wehrens & Buydens, 2007). The model was first constructed with 10,000 iterations until it went to an unfaltering stage. We then calculated the Mutual Information (MI) in python and computed similarity index with cy2 module (Graf‐Vlachy, 2022) to measure the random variations between ramets and genets. In machine learning, the MI between two random variables measures nonlinear relations between them and indicates how much information can be obtained from a random variable by observing another random variable. By comparing the epigenetic patterns between genets and ramets, five types of values for comparing including aHash, dHash, pHash, hist with split, and single hist were calculated.

Suitable areas for species restoration were chosen based on the genetic diversity and epigenetic variations level using R package prioritizr (Hanson et al., 2017). This package uses mixed integer linear programming (MILP) techniques to provide a flexible interface for building and solving restoration planning problems. We developed trade‐offs restoration prioritizations to identify priority areas for protected area establishment based on the degrees of which environmental variables contribute to genetic diversity and epigenetic variation. Our aim was to ensure that 20% of total genetic diversity and epigenetic variation among the surveyed populations are conserved (Failler et al., 2019). Environmental variables that correlated to genetic and epigenetic indices were used as penalty and constraint factors to solve the restoration planning problems.

## 3 RESULTS

### 3.1 Environmental gradient

**FIGURE 2:**
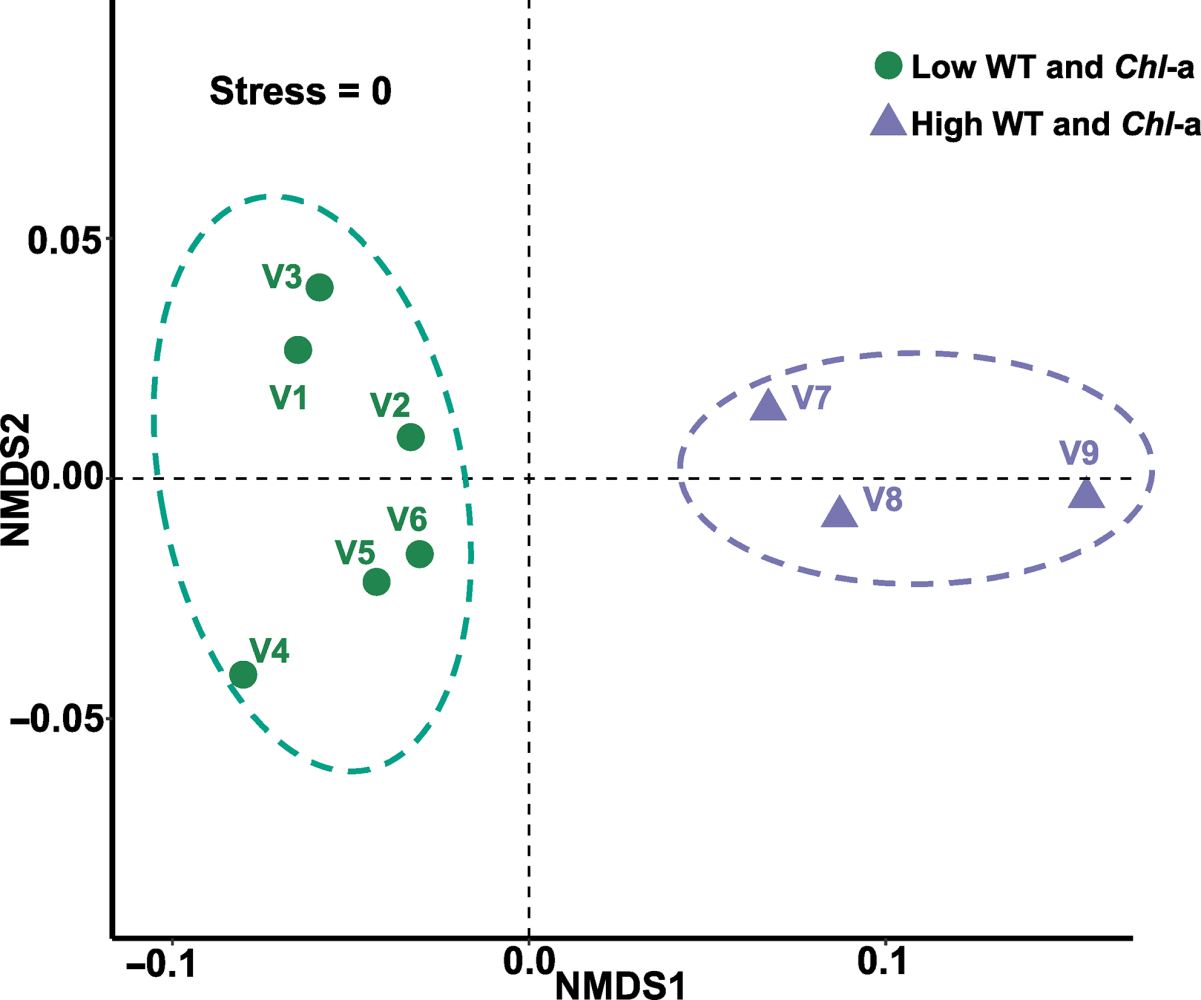
Results of NMDS reveal two environmental groups based on Euclidean distance at each population site of *Vallisneria natans*.

We captured a substantial environmental gradient over the sampling area (Stress = 0, Figure 2) according to the results of non‐metric multidimensional scaling (NMDS) analysis. In particularly, water body temperature and chlorophyll a shown an obvious gradient at a relatively fine geographic water body scale.

### 3.2 Genetic diversity and epigenetic variation

A total of 476 AFLP loci were obtained from nine populations of *V. natans* by using eight AFLP primers. The number of bands generated by different primer combinations ranged from 54 to 64 (Table S2). At species level, the mean Nei's diversity (*H*_E_), mean Shannon's information index (*I*), and mean percentage of polymorphic loci was 0.320%, 0.474%, and 85.93%, respectively. At the population level, the percentage of polymorphic bands (*PPL*) of each population varied from 79.93% (V9) to 92.02% (V5). Population V5 (*H*_E_ = 0.354, *I* = 0.522, *PPL* = 92.02%) exhibited the highest level of genetic diversity, and population V9 (*H*_E_ = 0.280, *I* = 0.419, *PPL* = 79.93%) showed the lowest level of genetic diversity (Table 1).

Based on five MSAP primer combinations, 392 epigenetic loci were produced, of which 74.2% (291 loci) were methylated loci. The percentage of methylation polymorphism among populations varied from 76.18% (V3) to 90.60% (V5 and V8), with an average of 83.91%. Population V5 demonstrated the highest levels of epigenetic diversity (mean e*H*_E_ = 0.315, e*I* = 0.474, e*PPL* = 90.60%) and population V7 (mean e*H*_E_ = 0.214, e*I* = 0.344, e*PPL* = 83.70%) showed the lowest level of epigenetic variation (Table 1, Table S3).

**FIGURE 3:**
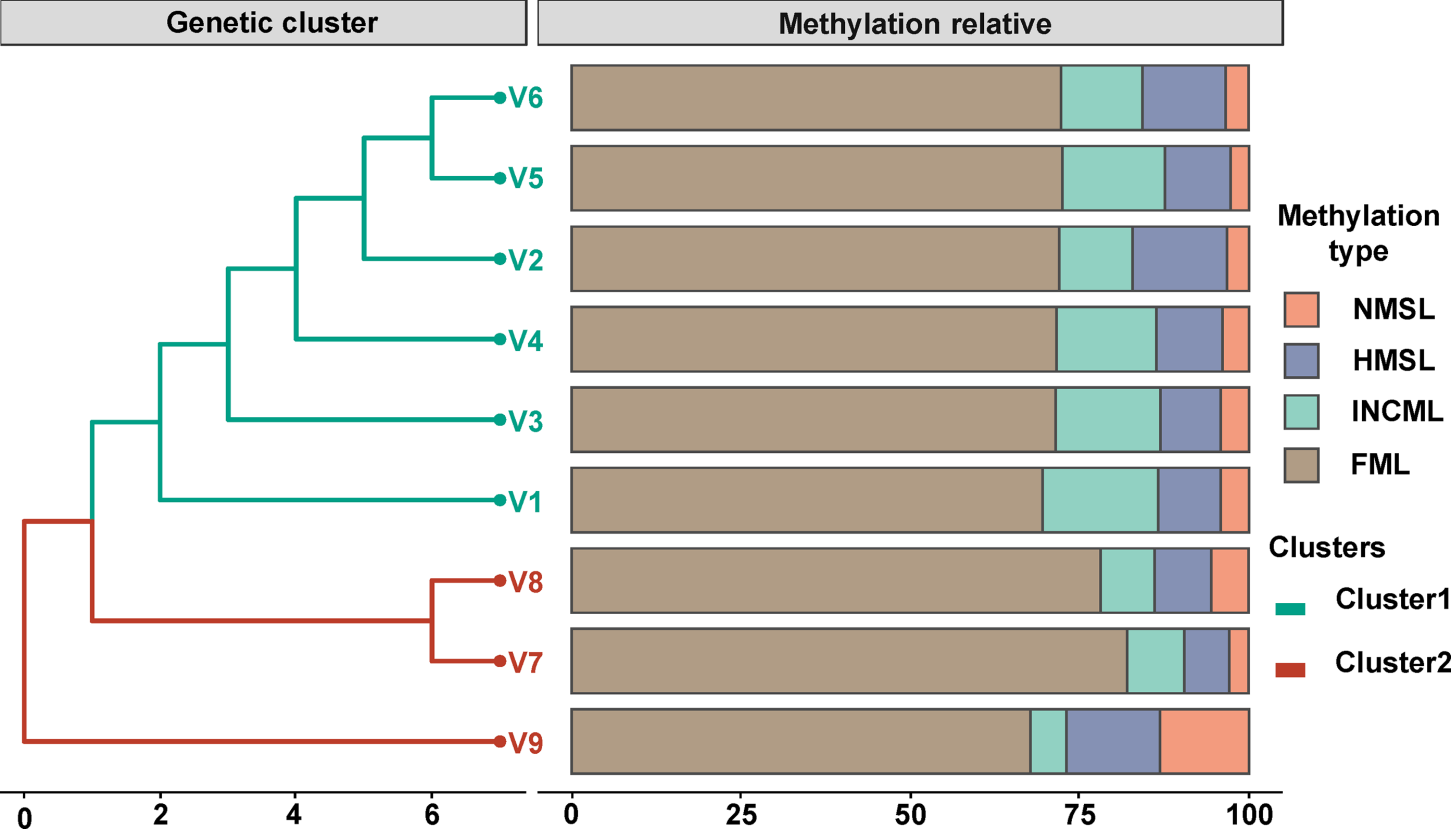
Dendrograms generated using unweight pair group method with arithmetic average, showing genetic relationships between nine populations of *Vallisneria natans* based on the Jaccard genetic similarity coefficient (left). Relative abundance of population‐based methylation among *V. natans* samples, NMSL represented non‐methylation, HMSL depicted the hemi‐methylated proportion, INCML indicated the inner cytosine methylation and FML was classified as full methylated state of DNA.

The population‐based UPGMA tree contains two genetic clades among *V. natans* populations based on the Jaccard genetic similarity coefficient (Figure 3). In addition, the methylation types of the loci varied among the genetic clades: populations V7, V8, and V9 exhibited lower levels of inner cytosine methylation types (mean INCML = 7.30%) than populations V1‐V6 (mean INCML = 14.23%). Non‐hierarchical analysis of molecular variance (AMOVA) of the genetic data revealed within‐population genetic differentiation of 87%, indicating strong genetic variation among populations. Hierarchical AMOVA attributed 6.9% of the total variance to differences between two groups and 8.5% to differences between groups among populations, whereas 84.7% variance was partitioned within populations (Table S4).

### 3.3 Population genetic structure

In the population structure analysis, the highest likelihood at *K* = 2 was revealed based on Bayesian assignment (Figure 1, Figure S1). Clades within the *V. natans* populations consisted of two clusters: populations from lower water body temperature and *Chl*‐a concentration areas (V7, V8, and V9) were classified as cluster 1, whereas populations from higher water body temperature and *Chl*‐a concentration areas (V1, V2, V3, V4, V5, and V6) were classified as cluster 2.

### 3.4 Correlations among genetic diversity, epigenetic variation, and environmental variables

**FIGURE 4:**
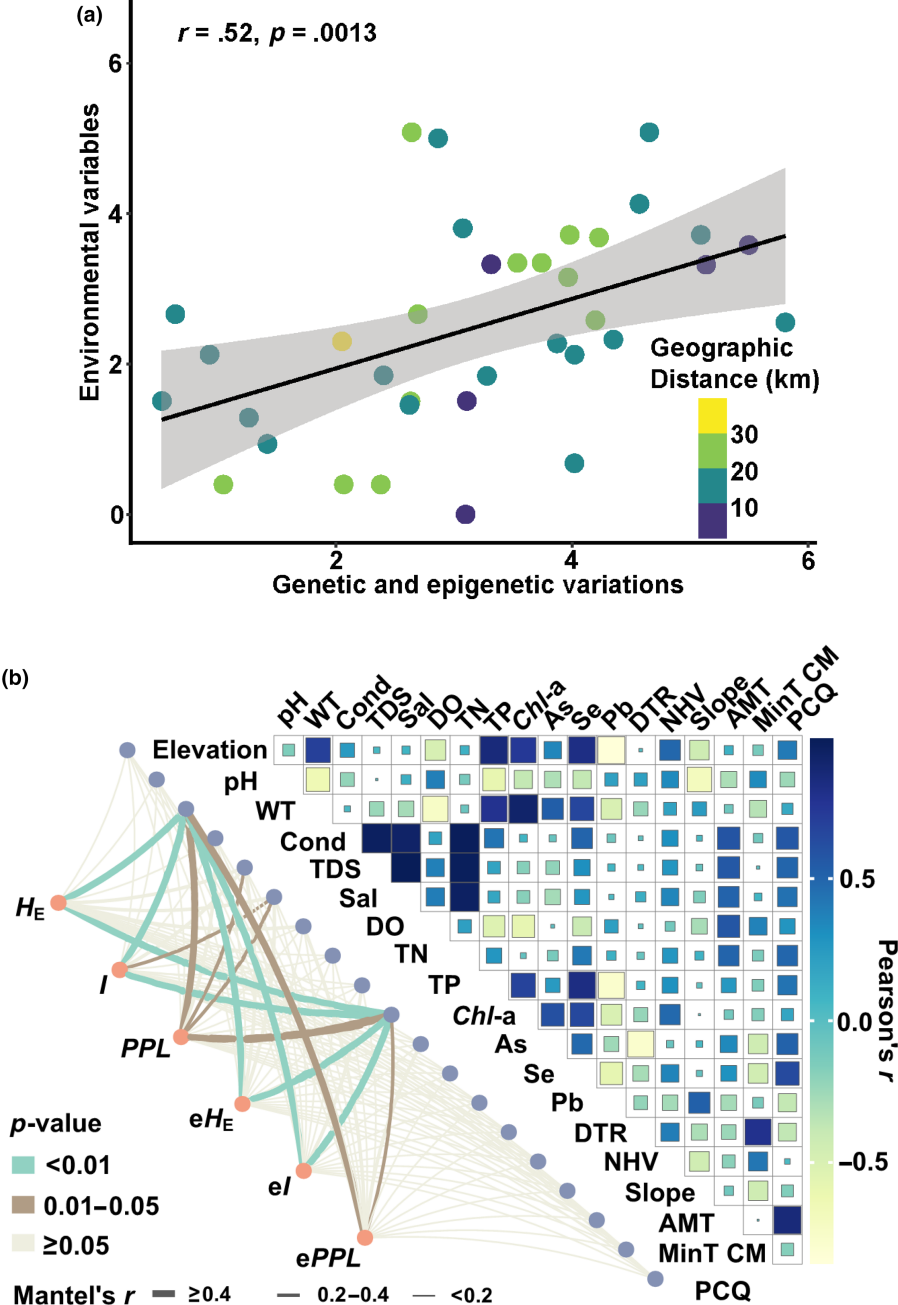
Correlations among genetic distance, geographic distance, and Euclidean environmental distance of nine *Vallisneria natans* populations. (a) Pearson correlations between genetic/epigenetic variables and environment variables, geographic distance among populations are shown in colored gradient plot. (b) Mantel test detected the coefficient of genetic diversity and epigenetic variation with environmental variables.

The Mantel test revealed a significant pattern of correlation between genetic and epigenetic variations with environmental variables (*r* = .52, *p* = .0013) (Figure 4). We filtered environmental variables that were obviously (MIC > 0.5) correlated with the genetic and epigenetic indices by using the MIC model (Figure S2b). In compliance with the Bvstep results and RF model (Tables S7 and S8, Figure S2a), 19 environmental variables were selected for analysis. Environmental variables including Sal, TDS, WT, and *Chl*‐a were strongly correlated with the genetic and epigenetic variation indices (Mantel's *r* > .2, *p* < .05) (Figure 4b). Particularly, genetic diversity (*H*_E_, *I*) and epigenetic variation (e*H*_E_, e*I*) indices presented an obvious correlation with WT and *Chl*‐a (Mantel's *r* > .4, *p* < .01).

Of the core variants detected by LFMM 2, six genetic loci were associated with environmental variables (FDR < 0.01) (Figure S3), indicating that adaptation to habitat heterogeneity has primarily evolved as a result of selection acting on regulatory environment changes. In particular, the three most significant epigenetic outliers were detected by LFMM 2 (FDR < 0.01), where locus2 was strongly associated with WT, TDS, Sal, and *Chl*‐a, locus245 was associated with TDS and Sal (Figure S4).

**FIGURE 5:**
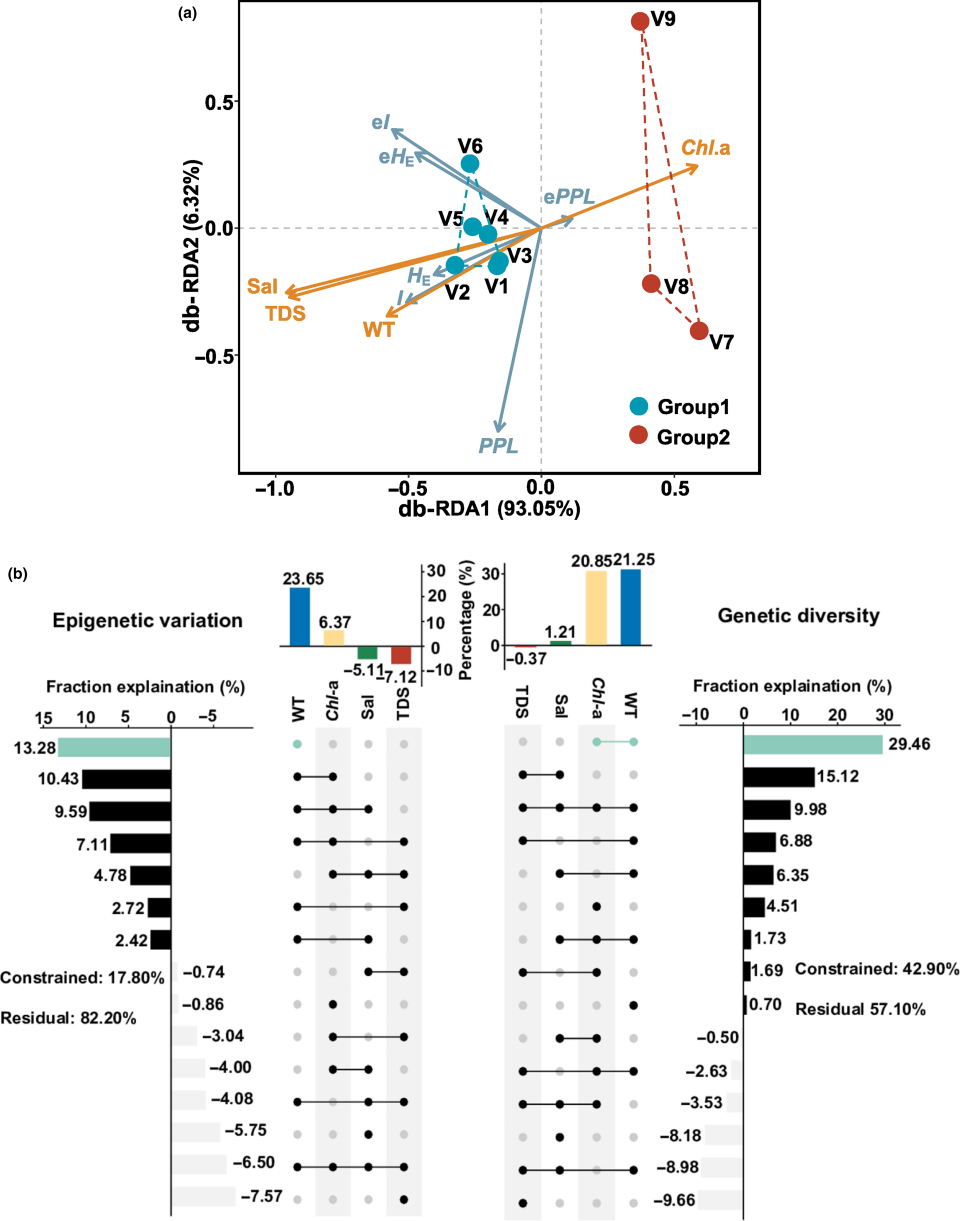
Distance‐based redundancy analysis plot demonstrated the ordination of genetic and epigenetic indices responses from *Vallisneria natans* under the influences of four environmental variables (a) and histogram of the relative contributions (b) of environmental variables in contrast to genetic diversity and epigenetic variation in Liangzi Lake. Sampled populations are colored points and points are enclosed by two different colors of lines in each genetic cluster. Vector in blue represent genetic and epigenetic indices and red arrows represent measured environmental variables. The numbers in the graphs are the percentage of variance explained. The dot matrix and the corresponding bar show the values of pure and shared contributions. Negative values due to adjustment of *R* ^2^ mean negligible but included in the computation of the total contribution of four environmental variables. Environmental variables with largest individual and overlapping contributions are highlighted in forest green. The residuals value represents the percentage of unexplained by the environmental variables.

In distance‐based redundancy analysis (db‐RDA), the first two axes explained 99.37% of the variances, with db‐RDA axes 1 and 2 accounted for 93.03% and 6.32% of the genetic and epigenetic variation, respectively (Figure 5a). The WT and *Chl*‐a presented a vital effect and had the largest contribution to genetic diversity and epigenetic variation (fraction of effect: 21.25% and 23.65%, respectively). In fact, WT, *Chl*‐a shared the effects on genetic diversity (combined effect = 29.46%) through variance partitioning analysis, WT exhibits the highest individual effect on epigenetic variation through hierarchical partitioning (individual effect = 13.28%) (Figure 5b).

**FIGURE 6:**
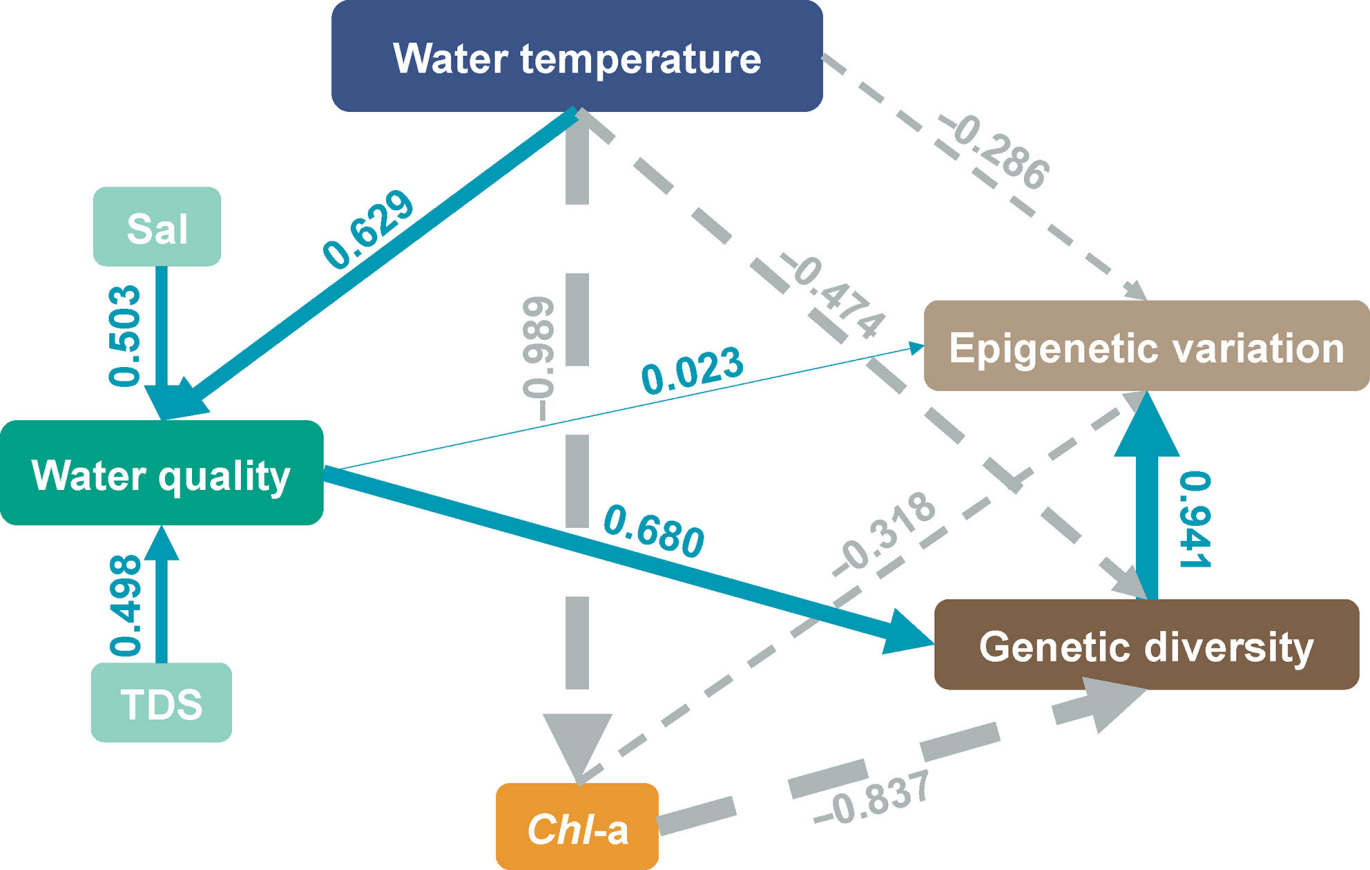
Effects of water temperature, water quality, and *Chl*‐a on genetic diversity and epigenetic variation. Water quality is a latent variable coupled with total dissolved solids and salinity. The line width is proportional to the effect strength. Numbers adjacent to arrows are standardized path coefficients. Continuous and dashed arrows indicate positive and negative relationships, respectively.

Our PLS‐PM model showed the robustness of our data, as suggested by the goodness of fit (GoF = 0.91). The PLS‐PM analysis determined that WT had a negative influence on variation, accounting for 65.8% (*R* ^2^ = .658) of variation in genetic diversity (path coefficient = −0.474, *p* < .05) and epigenetic variation (path coefficient = −0.286, *p* < .05) (Figure 6). *Chl*‐a also exerted negative effects on genetic and epigenetic variations (path coefficient: −0.318 and −0.837, *p* < .05, respectively). However, water quality had positive effects on genetic diversity (path coefficient = 0.023, *p* < .05) and epigenetic variations (path coefficient = 0.680, *p* < .05). A strong impact was detected from genetic diversity to epigenetic variation (path coefficient = 0.941, *p* < .05).

### 3.5 Variations between genets and ramets

**FIGURE 7:**
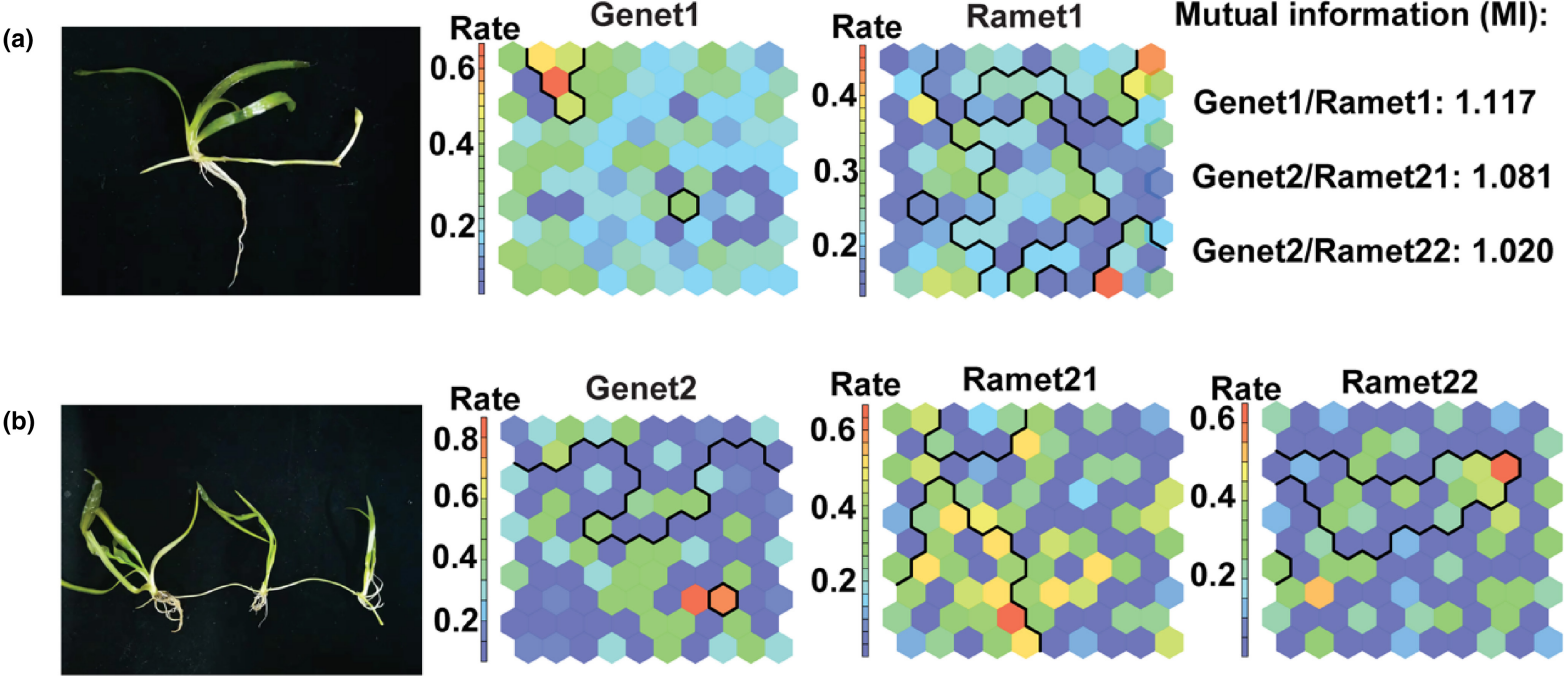
Methylation variation between genets and ramets in self‐organizing map clustering results. (a) Variation patterns between in 22 *Vallisneria natans* individuals. (b) Methylated locus varied from genets to the first and second clonal generations in eight *V. natans* individuals.

The SOM cluster showed the changing loci from the genets to ramets with varied methylated rates (Figure 7). Based on the MI results, in the 22 *V. natans* genets and first clonal individuals, the clonal ramets showed epigenetic variations compared with the genets (MI = 1.117). However, in eight *V. natans* genets and second clonal individuals, although the first clonal generations variated from genets (MI = 1.081), the second clonal generations depicted lower epigenetic variation rates (MI = 1.020) than the first clonal generations. These finds indicated an ancestral scenario.

Among the five comparison values in the analysis, aHash, dHash, Hist with split, and Single Hist were consistent at the methylated locus level between genets and the second clonal generations (Table S6). The values between genets and first clonal ramets were 0.85, 0.82, 0.49, and 0.44, while the values between genets and the second clonal ramets were higher (0.89, 0.86 0.56, and 0.49, respectively). Higher values indicated more similarity between two epigenetic variation patterns, consistent with the MI analysis.

From the aspect of different methylation types, the 22 ramets did not show devastating variations, with 80.0% identity of NMSL, 90.0% of HEML, 100% of INCML, and 97.3% of FML (Figure S5a). However, in 8 second clonal generations, the first clonal individuals of NMSL, INCML, HMSL, and FML variated considerably in comparison with the genets (89.4%, 88.7%, 77.2%, and 93.9%). Based on the comparison between the second generations and the genets, both indices showed little divergence (INCML = 82.1%, HEML = 91.6%, FML = 98.0%) (Figure S5b).

### 3.6 Species restoration areas

**FIGURE 8:**
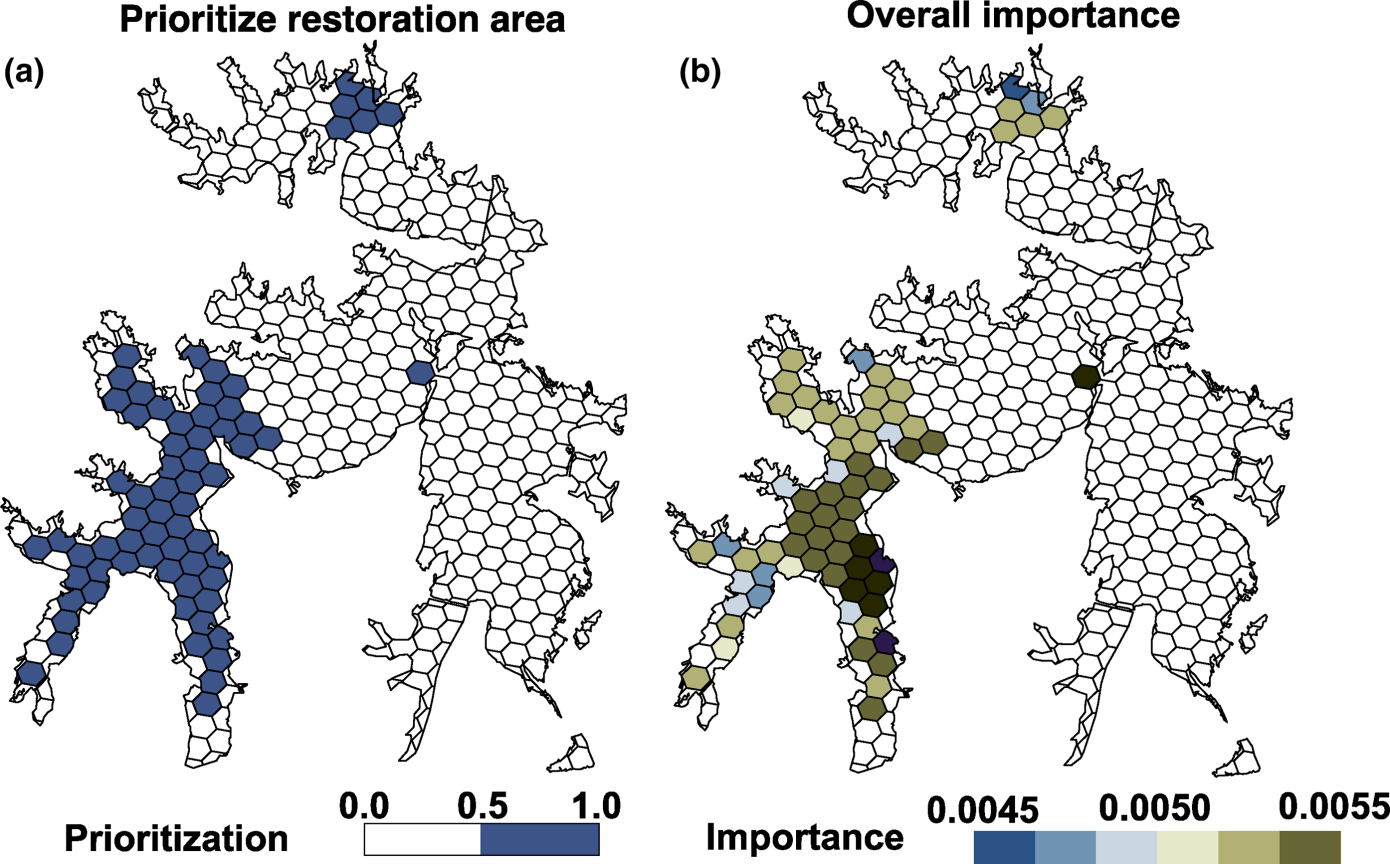
Conserved units including 20% of the species' genetic diversity and epigenetic variation. *Chl*‐a is used as environmental constraint factors to fulfill the restoration requirement. Blue hexagons refer to prioritize restoration area (a), and the relative importance was shown in gradient color ramps (b).

The lake area was divided into priority and overall important areas for species restoration based on genetic diversity and epigenetic variation (Figure 8). Due to the correlations in WT, *Chl*‐a, genetic variation, and epigenetic variations, the restoration patterns are constructed based not only on the levels of genetic and epigenetic variation but also on environmental variables. Therefore, both WT and *Chl*‐a were used as constraint factors during the construction of restoration patches.

## 4 DISCUSSION

### 4.1 Genetic diversity and epigenetic variation

Aquatic plants with sexual production tend to harbor high genetic diversity as their seed could be dispersed by water fluent, leading to an equilibrium pattern of genetic variations in fine‐scale areas (Honnay et al., 2010). However, the dynamics of habitats usually force species to compensate the rapid changes as plants are usually rooted in soil (Farleigh et al., 2021; Ortego et al., 2012). The response of aquatic species under changing environmental conditions is exclusively based on genetic and epigenetic variation (Gao et al., 2022). Our results showed high genetic and epigenetic variations in *V. natans* populations under WT and *Chl*‐a gradient (mean *PPL* = 85.93%, mean e*PPL* = 83.91%), consistent with a previous genetic study using ISSR markers of the same genera (52%–62% *PPL*) (Wang et al., 2020). We further detected a strong correlation between genetic diversity and epigenetic variation, indicating their association during the evolution process linking of *V. natans* populations.

The surroundings of water body play an important role in aquatic plant growth and propagation. Environmental oscillations may deeply influence the species' genetic diversity and epigenetic variations (Verhoeven et al., 2010). Empirical studies on aquatic plants indicated that environmental variables could influence the genotypic diversity and plants growth (Ghosh et al., 2022; Hughes & Stachowicz, 2009). Considering that measurement the surrounding environmental variables of species habitats is a seemingly simple yet daunting task, correlation tests remain lacking in environmental‐based genetic and epigenetic study. In our study, a profound positive correlation between genetic/epigenetic variation and environmental variables was found. High genetic and epigenetic variations were also detected to be correlated with lower water temperature and *Chl*‐a concentration. Meanwhile, genetic and epigenetic loci were found to associated with environmental variables tested. Consistent with our similar work on *Ceratophyllum demersum* at the same location (Y. Li, X. Zhao, M. Xia, H. Hou, unpublished data), high genetic and epigenetic variations were found to be correlated with low temperatures. By using the published data, we detected negative correlations between annual mean temperature and the genetic diversity in *Hydrocotyle vulgaris* (Wang et al., 2020). This finding confirmed the hypothesis that environmental gradient drives the genetic and epigenetic variations in aquatic plants.

### 4.2 Genetic structure

In aquatic systems, submerged plants with less migration rates usually maintain more genetic structure because asymmetric gene flow is expected to be confined to a fine scale, thereby reducing genetic interaction in some isolated areas (Pollux et al., 2007). However, a recent study revealed that other than the geographic features, anthropogenic stressors, especially occurrence of environmental chemical stresses, can shape the genetic structure in aquatic systems (Inostroza et al., 2016).

One of the questions raised by our study is whether the strong genetic structure is driven by geographic isolation or environmental factors. The answer is that environment factors may contribute to the occurrence status of genetic structure of *V. natans*. Early reports indicated that *V. natans* are divided into local adaptive sub‐species by differential geographical barriers (Cao et al., 2021). Our sample area covered the full range of the lake area but different seasons; however, the population structure of *V. natans* does not show genetic differentiation from a geographic isolation but demonstrates a fiercely separation along temperature and *Chl*‐a gradient. As stated above, the genetic diversity of *V. natans* is negatively correlated with water body temperature and *Chl*‐a, and genetic distance is not correlated with genetic diversity. This finding is in agreement with our analysis in another aquatic herb *Ceratophyllum demersum* (Y. Li, X. Zhao, M. Xia, H. Hou, unpublished data), of which temperature promotes the shaping of the genetic structure.

### 4.3 Epigenetic variations between genets and ramets

Multicellular organisms are genetic mosaics owing to their somatic mutations. Somatic genetic variation may play a different, more positive role in multicellular species undergoing clonal reproduction (Yu et al., 2020). Populations of ramets collectively form clone or genets through a single sexually produced genotype; importantly, when a clonal offspring is produced, somatic genetic variation may be unequally distributed (Wibowo et al., 2018). Previous epigenetic study on dandelion revealed that most methylation changes could be transmitted from genets to offspring (between 74% and 92% of the changes); only a small proportion reverted to the consensus epigenotype (Preite et al., 2015; Verhoeven et al., 2010). Consistently, the present results indicated that the first clonal generations had little variations than the genets. As epigenetic variations play an independent role in evolutionary process of dandelion, the strong correlation between genetic diversity and epigenetic variation in our analysis may influence the epigenetic variation process between genets and the first clonal generations.

As a prominent epigenetic modification, cytosine methylation may play a critical role in the adaptation of plants to different environments (Eckert et al., 2021). Although DNA methylation is mainly static, the landscape of methylation does vary across generations (Skipper, 2011). In our epigenetic analysis of eight clonal generation lines, the INCML, HMSL, and FML in the first clonal generations varied significantly, while the genets and the second clonal generations were identical. By studying genets‐ramets epigenetic variation, our full DNA methylation analysis revealed that the first clonal generation maintained a relative higher level of methylation rate than the genets. However, an atavism scenario was shown in the second generation, which depicted a lower level of methylation compared with that in the first clonal generation. A previous study confirmed that gene function could not inherited stably under a methylated stage but can be re‐activated in second generation (Li et al., 2020). Our study may sustain the point and notion the epigenetic variations fluctuate between generations and that the process involves an ancestral scenario.

### 4.4 Implications for restoration

Genetic diversity and epigenetic variations usually reflect the capacity of species adaptation during evolution and should be consideration when designing restoration sites (Teixeira & Huber, 2021). With regard to aquatic species restoration, limited information is known about genetic variation and species–sites interactions, especially for restoration projects aimed at mitigating the effects of extreme climate events or anthropogenic factors (Forester et al., 2022). Furthermore, epigenetic variations are crucial for the adaptation of species to unforeseen changes in climate and for maintaining resilience to abiotic and biotic stresses. The Liangzi Lake is a protected area buffered from anthropogenic threats. As protected areas should conserve not only intraspecific genetic diversity but also habitat heterogeneity, identifying potential relationships between genetic and epigenetic variation and environmental variables and linking these relationships to fitness would offer opportunity to construct restoration portfolios even when environmental pressures are difficult to measure (Bay et al., 2017). Based on our analysis, the suitability of areas for species restorations depends not only on the levels of genetic diversity and epigenetic variations but also on incremental temperature benefits. Trade‐offs must be made among genetic diversity, epigenetic variation, water body temperature, and *Chl*‐a concentrations when designing prioritized restoration areas.

## 5 CONCLUSIONS

The patterns of genetic diversity and epigenetic variations in *V. natans* reflect the population history and recent habitats changes. In line with our genetic data, *V. natans* is potentially sensitive to the ever‐changing environment. The distribution of genetic and epigenetic variations emphasized the importance of having better understanding of the environmental variables that may affect variations in this macrophyte species. The current status level of genetic diversity seems to be related to environmental factors. Therefore, environmental variables should not be neglected during genetic and epigenetic analyses in aquatic systems, especially for the restoration purpose. Additionally, our study presents the broad scenario of species adaption and depicts novel insights into epigenetic variations in asexual plants, especially in aquatic plants.

## AUTHOR CONTRIBUTIONS

**Yixian Li:** Conceptualization (lead); data curation (lead); formal analysis (lead); investigation (lead); methodology (lead); resources (lead); software (lead); visualization (lead); writing – original draft (lead); writing – review and editing (supporting). **Xuyao Zhao:** Formal analysis (supporting); resources (supporting); writing – review and editing (supporting). **Manli Xia:** Investigation (supporting); methodology (supporting). **Hongwei Hou:** Conceptualization (lead); funding acquisition (lead); project administration (lead); resources (lead); supervision (lead); validation (lead); writing – review and editing (lead).

## FUNDING INFORMATION

Funding was provided by the National Key R & D Program of China (2018YFD0900801).

### OPEN RESEARCH BADGES

This article has earned Open Data, Open Materials and Preregistered Research Design badges. Data, materials and the preregistered design and analysis plan are available at https://github.com/yixian185/data‐for‐ECE.

## Supporting information


Appendix S1

## Data Availability

The datasets used in this study are freely available from the follow‐ing sources: Codes and data are openly available from the Github repository: https://github.com/yixian185/data‐for‐ECE; Bioclimatic data and digital elevation model: WorldClim 2.1 (https://www.worldclim.org).

## References

[ece310434-bib-0001] Bay, R. A. , Rose, N. , Barrett, R. , Bernatchez, L. , Ghalambor, C. K. , Lasky, J. R. , Brem, R. B. , Palumbi, S. R. , & Ralph, P. (2017). Predicting responses to contemporary environmental change using evolutionary response architectures. The American Naturalist, 189, 463–473. 10.1086/691233 28410032

[ece310434-bib-0002] Cao, Q. J. , Liu, B. B. , & Hu, F. Y. (2021). Effects of hydrological connection and human disturbance on genetic variation of submerged *Vallisneria natans* populations in four lakes in China. Journal of Oceanology and Limnology, 39, 1403–1416.

[ece310434-bib-0003] Caye, K. , Jumentier, B. , Lepeule, J. , & François, O. (2019). LFMM 2: Fast and accurate inference of gene‐environment associations in genome‐wide studies. Molecular Biology and Evolution, 36, 852–860. 30657943 10.1093/molbev/msz008 PMC6659841

[ece310434-bib-0004] Crane, K. , Kregting, L. , Coughlan, N. E. , Cuthbert, R. N. , Ricciardi, A. , MacIsaac, H. J. , Dick, J. T. A. , & Reid, N. (2022). Abiotic and biotic correlates of the occurrence, extent and cover of invasive aquatic *Elodea nuttallii* . Freshwater Biology, 67, 1559–1570. 36246039 10.1111/fwb.13960 PMC9545499

[ece310434-bib-0005] Earl, D. A. , & VonHoldt, B. M. (2012). STRUCTURE HARVESTER: A website and program for visualizing STRUCTURE output and implementing the Evanno method. Conservation Genetics Resources, 4, 359–361.

[ece310434-bib-0006] Eckert, S. , Herden, J. , Stift, M. , Joshi, J. , & van Kleunen, M. (2021). Manipulation of cytosine methylation does not remove latitudinal clines in two invasive goldenrod species in Central Europe. Molecular Ecology, 30, 222–236. 33150604 10.1111/mec.15722

[ece310434-bib-0007] Engelhardt, K. A. M. , Lloyd, M. W. , & Neel, M. C. (2014). Effects of genetic diversity on conservation and restoration potential. at individual, population, and regional scales. Biological Conservation, 179, 6–16.

[ece310434-bib-0008] Failler, P. , Touron‐Gardic, G. , & Traore, M. S. (2019). Is Aichi target 11 progress correctly measured for developing countries? Trends in Ecology & Evolution, 34, 875–879. 31395290 10.1016/j.tree.2019.07.007

[ece310434-bib-0009] Farleigh, K. , Vladimirova, S. A. , Blair, C. , Bracken, J. T. , Koochekian, N. , Schield, D. R. , Card, D. C. , Finger, N. , Henault, J. , & Leaché, A. D. (2021). The effects of climate and demographic history in shaping genomic variation across populations of the Desert Horned Lizard (*Phrynosoma platyrhinos*). Molecular Ecology, 30, 4481–4496. 34245067 10.1111/mec.16070

[ece310434-bib-0010] Filosi, M. , Visintainer, R. , Albanese, D. , Riccadonna, S. , Jurman, G. , Furlanello, C. , & Filosi, M. M. (2012). Package ‘minerva’ .

[ece310434-bib-0011] Forester, B. R. , Beever, E. A. , Darst, C. , Szymanski, J. , & Funk, W. C. (2022). Linking evolutionary potential to extinction risk: Applications and future directions. Frontiers in Ecology and the Environment, 20, 507–515.

[ece310434-bib-0012] Fu, F. C. , Huang, S. B. , Yuan, J. Q. , Du, Z. L. , Cao, Y. , Yuan, H. G. , Zhou, W. J. , Wu, J. H. , Yi, H. L. , Chen, B. B. , & Zhang, Y. Q. (2021). Effect evaluation of remediation in three typical rivers with *Vallisneria natans* under different pollution and spatial characterization of functional microorganisms. Ecological Engineering, 171, 12.

[ece310434-bib-0013] Gao, Y. , Chen, Y. , Li, S. , Huang, X. , Hu, J. , Bock, D. G. , MacIsaac, H. J. , & Zhan, A. (2022). Complementary genomic and epigenomic adaptation to environmental heterogeneity. Molecular Ecology, 31, 3598–3612. 35560847 10.1111/mec.16500

[ece310434-bib-0014] Ghosh, D. , Sarkar, A. , Basu, A. G. , & Roy, S. (2022). Effect of plastic pollution on freshwater flora: A meta‐analysis approach to elucidate the factors influencing plant growth and biochemical markers. Water Research, 225, 119114. 36152443 10.1016/j.watres.2022.119114

[ece310434-bib-0015] Graf‐Vlachy, L. (2022). CV2: Stata module to calculate the coefficient of variation for variables .

[ece310434-bib-0016] Greig, H. S. , Kratina, P. , Thompson, P. L. , Palen, W. J. , Richardson, J. S. , & Shurin, J. B. (2012). Warming, eutrophication, and predator loss amplify subsidies between aquatic and terrestrial ecosystems. Global Change Biology, 18, 504–514.

[ece310434-bib-0017] Hanson, J. , Schuster, R. , Morrell, N. , Strimas‐Mackey, M. , Watts, M. , Arcese, P. , Bennett, J. , & Possingham, H. (2017). prioritizr: Systematic conservation prioritization in R . R package version 3. 10.1111/cobi.14376 PMC11780203 39268847

[ece310434-bib-0018] Honnay, O. , Jacquemyn, H. , Nackaerts, K. , Breyne, P. , & Van Looy, K. (2010). Patterns of population genetic diversity in riparian and aquatic plant species along rivers. Journal of Biogeography, 37, 1730–1739.

[ece310434-bib-0019] Hughes, A. R. , & Stachowicz, J. J. (2009). Ecological impacts of genotypic diversity in the clonal seagrass *Zostera marina* . Ecology, 90, 1412–1419. 19537560 10.1890/07-2030.1

[ece310434-bib-0020] Hulce, D. , Li, X. , Snyder‐Leiby, T. , & Liu, C. J. (2011). GeneMarker® genotyping software: Tools to increase the statistical power of DNA fragment analysis. Journal of Biomolecular Techniques, 22, S35.

[ece310434-bib-0021] Inostroza, P. A. , Vera‐Escalona, I. , Wicht, A.‐J. , Krauss, M. , Brack, W. , & Norf, H. (2016). Anthropogenic stressors shape genetic structure: Insights from a model freshwater population along a land use gradient. Environmental Science & Technology, 50, 11346–11356. 27643810 10.1021/acs.est.6b04629

[ece310434-bib-0022] Kamvar, Z. N. , Tabima, J. F. , & Grünwald, N. J. (2014). Poppr: An R package for genetic analysis of populations with clonal, partially clonal, and/or sexual reproduction. PeerJ, 2, e281. 24688859 10.7717/peerj.281 PMC3961149

[ece310434-bib-0023] Kursa, M. B. , Rudnicki, W. R. , & Kursa, M. M. B. (2020). Package ‘Boruta’ .

[ece310434-bib-0024] Lai, J. , Zou, Y. , Zhang, J. , & Peres‐Neto, P. R. (2022). Generalizing hierarchical and variation partitioning in multiple regression and canonical analyses using the rdacca. hp R package. Methods in Ecology and Evolution, 13, 782–788.

[ece310434-bib-0025] Leitch, A. R. , Leitch, I. J. , Trimmer, M. , Guignard, M. S. , & Woodward, G. (2014). Impact of genomic diversity in river ecosystems. Trends in Plant Science, 19, 361–366. 24447819 10.1016/j.tplants.2013.12.005

[ece310434-bib-0026] Li, J. , Yang, D.‐L. , Huang, H. , Zhang, G. , He, L. , Pang, J. , Lozano‐Durán, R. , Lang, Z. , & Zhu, J.‐K. (2020). Epigenetic memory marks determine epiallele stability at loci targeted by de novo DNA methylation. Nature Plants, 6, 661–674. 32514141 10.1038/s41477-020-0671-x

[ece310434-bib-0027] Oksanen, J. , Kindt, R. , Legendre, P. , O'Hara, B. , Stevens, M. H. H. , Oksanen, M. J. , & Suggests, M. (2007). The vegan package. Community ecology package, 10, 719–3223.

[ece310434-bib-0028] Ortego, J. , Riordan, E. C. , Gugger, P. F. , & Sork, V. L. (2012). Influence of environmental heterogeneity on genetic diversity and structure in an endemic southern Californian oak. Molecular Ecology, 21, 3210–3223. 22548448 10.1111/j.1365-294X.2012.05591.x

[ece310434-bib-0029] Paradis, E. , & Schliep, K. (2019). Ape 5.0: An environment for modern phylogenetics and evolutionary analyses in R. Bioinformatics, 35, 526–528. 30016406 10.1093/bioinformatics/bty633

[ece310434-bib-0030] Pérez‐Figueroa, A. (2013). msap: A tool for the statistical analysis of methylation‐sensitive amplified polymorphism data. Molecular Ecology Resources, 13, 522–527. 23311622 10.1111/1755-0998.12064

[ece310434-bib-0031] Pollux, B. , Jong, M. , Steegh, A. , Verbruggen, E. , Van Groenendael, J. , & Ouborg, N. (2007). Reproductive strategy, clonal structure and genetic diversity in populations of the aquatic macrophyte *Sparganium emersum* in river systems. Molecular Ecology, 16, 313–325. 17217347 10.1111/j.1365-294X.2006.03146.x

[ece310434-bib-0032] Preite, V. , Snoek, L. B. , Oplaat, C. , Biere, A. , van der Putten, W. H. , & Verhoeven, K. J. (2015). The epigenetic footprint of poleward range‐expanding plants in apomictic dandelions. Molecular Ecology, 24, 4406–4418. 26206253 10.1111/mec.13329

[ece310434-bib-0033] Pritchard, J. K. , Stephens, M. , & Donnelly, P. (2000). Inference of population structure using multilocus genotype data. Genetics, 155, 945–959. 10835412 10.1093/genetics/155.2.945 PMC1461096

[ece310434-bib-0034] Ravand, H. , & Baghaei, P. (2016). Partial least squares structural equation modeling with R. Practical Assessment, Research and Evaluation, 21, 11.

[ece310434-bib-0035] Robertson, M. , Schrey, A. , Shayter, A. , Moss, C. J. , & Richards, C. (2017). Genetic and epigenetic variation in *Spartina alterniflora* following the Deepwater Horizon oil spill. Evolutionary Applications, 10, 792–801. 29151871 10.1111/eva.12482 PMC5680422

[ece310434-bib-0036] Schulz, B. , Eckstein, R. L. , & Durka, W. (2014). Epigenetic variation reflects dynamic habitat conditions in a rare floodplain herb. Molecular Ecology, 23, 3523–3537. 24943730 10.1111/mec.12835

[ece310434-bib-0037] Skipper, M. (2011). Epigenetic variation across the generations. Nature Reviews Genetics, 12, 740. 10.1038/nrg3084 21989131

[ece310434-bib-0038] Smouse, R. P. P. , & Peakall, R. (2012). GenAlEx 6.5: Genetic analysis in Excel. Population genetic software for teaching and research—An update. Bioinformatics, 28, 2537–2539. 22820204 10.1093/bioinformatics/bts460 PMC3463245

[ece310434-bib-0039] Taylor, M. (2017). sinkr: Collection of functions with emphasis in multivariate data analysis . R package version 0.6.

[ece310434-bib-0040] Teixeira, J. C. , & Huber, C. D. (2021). The inflated significance of neutral genetic diversity in conservation genetics. Proceedings of the National Academy of Sciences of the United States of America, 118, e2015096118. 33608481 10.1073/pnas.2015096118 PMC7958437

[ece310434-bib-0041] Verhoeven, K. J. F. , Jansen, J. J. , van Dijk, P. J. , & Biere, A. (2010). Stress‐induced DNA methylation changes and their heritability in asexual dandelions. New Phytologist, 185, 1108–1118. 20003072 10.1111/j.1469-8137.2009.03121.x

[ece310434-bib-0042] Verhoeven, K. J. F. , Vonholdt, B. M. , & Sork, V. L. (2016). Epigenetics in ecology and evolution: What we know and what we need to know. Molecular Ecology, 25, 1631–1638. 26994410 10.1111/mec.13617

[ece310434-bib-0043] Wang, B. , Song, Z. P. , Liu, G. H. , Lu, F. , & Li, W. (2010). Comparison of the extent of genetic variation of *Vallisneria natans* and its sympatric congener *V. spinulosa* in lakes of the middle‐lower reaches of the Yangtze River. Aquatic Botany, 92, 233–238.

[ece310434-bib-0044] Wang, M. Z. , Li, H. L. , Li, J. M. , & Yu, F. H. (2020). Correlations between genetic, epigenetic and phenotypic variation of an introduced clonal herb. Heredity, 124, 146–155. 31431739 10.1038/s41437-019-0261-8 PMC6906319

[ece310434-bib-0045] Waycott, M. (1998). Genetic variation, its assessment and implications to the conservation of seagrasses. Molecular Ecology, 7, 793–800.

[ece310434-bib-0046] Wehrens, R. , & Buydens, L. M. (2007). Self‐and super‐organizing maps in R: The Kohonen package. Journal of Statistical Software, 21, 1–19.

[ece310434-bib-0047] Wibowo, A. , Becker, C. , Durr, J. , Price, J. , Spaepen, S. , Hilton, S. , Putra, H. , Papareddy, R. , Saintain, Q. , & Harvey, S. (2018). Partial maintenance of organ‐specific epigenetic marks during plant asexual reproduction leads to heritable phenotypic variation. Proceedings of the National Academy of Sciences of the United States of America, 115, E9145–E9152. 30201727 10.1073/pnas.1805371115 PMC6166847

[ece310434-bib-0048] Yeh, F. C. (1999). POPGENE (version 1.3. 1) . Microsoft Window‐Bases Freeware for Population Genetic Analysis. http://www.ualberta.ca/~fyeh/

[ece310434-bib-0049] Yu, L. , Bostrom, C. , Franzenburg, S. , Bayer, T. , Dagan, T. , & Reusch, T. B. H. (2020). Somatic genetic drift and multilevel selection in a clonal seagrass. Nature Ecology & Evolution, 4, 952–962. 32393866 10.1038/s41559-020-1196-4

[ece310434-bib-0050] Zhou, Y. , Li, X. J. , Zhao, Y. , Zhou, W. , Li, L. , Wang, B. , Cui, X. H. , Chen, J. K. , & Song, Z. P. (2016). Divergences in reproductive strategy explain the distribution ranges of *Vallisneria* species in China. Aquatic Botany, 132, 41–48.

