# Supplementary material for: Water temperature and chlorophyll a density drive the genetic and epigenetic variation of Vallisneria natans across a subtropical freshwater lake
Source: Ecol Evol. 2023 Aug 15;13(8):e10434. doi: 10.1002/ece3.10434 (PMC10425707; doi:10.1002/ece3.10434)
Supplement: Supplementary file 1 — Appendix S1 [file ECE3-13-e10434-s001.docx]

**Supporting Information**

**Detailed Methods**

**AFLP and MSAP genotyping**

AFLP and MSAP genotyping were carried out according to the protocols recommended by Vos and modified by Baurens (Vos et al., 1995; Causse, & Legavre, 2008). For the AFLP analysis, 100 ng DNA in a total volume of 40 μL was digested using a cocktail composed of 0.15 μL *EcoR*I (20 U·µL^-1^) and 0.3 μL *Mse*I (20 U·µL^-1^) (New England Biolabs, Ipswich, MA, USA) for 1.5 hr at 37 ℃. For MSAP analysis, the DNA template was digested using *EcoR*I and a pair of isoschizomeric enzymes in place of *Mse*I: *Hpa*II and *Msp*I. Both of these enzymes recognize the CCGG sequence but show different sensitivities to the methylation status of external or internal cytosine residues, thereby reflecting the types of methylation based on the the lengths of the digested bands.

The products were visualized by 1.5% agarose gel electrophoresis to ensure that the templates were completely digested. Subsequently, 10 µL of a mixture which containing 3 µL *EcoR*I adaptor (5 µM), 3 µL *Mse*I/*Hpa*II/*Msp*I adaptor (50 µM), 1 µL T4 ligase buffer (10×), 0.5 µL T4 DNA ligase (400 U·µL^-1^) (New England Biolabs, Ipswich, USA), and 2.5 µL dd H_2_O was added to the samples. The ligation reaction was incubated overnight at 4 ℃ in an ABI Veriti 96-well thermocycler (Thermo Fisher Scientific, Waltham, MA, USA), and the products were diluted 10-fold in dd H_2_O for the amplification reaction.

For pre-selective DNA amplification, 5 µL of the diluted DNA restriction-ligation product was added to a 20 µL cocktail containing 2 µL 10×Ex-Taq buffer, 1.2 µL Mg^2+^ (25 mM), 1.6 µL dNTPs (2.5 mM), 1 µL primer (10 µM·L^-1^) E+A (Invitrogen, CA, USA), 1 µL primer (10 µM·L^-1^) M+C/T (Invitrogen, CA, USA), and 0.1 µL Ex-Taq (5 U·µL^-1^). PCR was performed at 65 °C for 5 min, followed by 30 cycles of 30 s of denaturation at 94 °C, 30 s of annealing at 56 °C, and 1 min of elongation at 72 °C, and a final step of 5 min at 72 °C step for complete extension. For selective PCR amplification, 25 µL 10-fold diluted pre-PCR product was added to a 15 µL cocktail containing 2 µL 10× Ex-Taq buffer, 2 µL Mg^2+^ (25 mM), 1.6 µL dNTPs (2.5 mM), 0.2 µL 5-FAM**EcoR*I (10 µM·L^-1^), 1.2 µL *Mse*I/*Hpa*II/*Msp*I (10 µM·L^-1^), and 0.1 µL Ex-Taq (5 U·µL^-1^). The PCR parameters were 2 min at 94 °C, 13 cycles of 30 s of denaturation at 94 °C, 30 s of annealing at 65 °C, and 1 min of elongation at 72 °C, but with the annealing temperature reduced every by 0.7 °C at each step; this was followed by an additional 23 cycles of 30 s of denaturation at 94°C, 30 s of annealing at 56°C, and 1 min of elongation at 72 °C, followed by a 5-min step at 72 °C. All reagents for PCR amplification were from Takara (Takara Biomedical, Beijing, China).

**Table S1. Environmental variables used in the assessment of habitat heterogeneity**

| **Name** | **Abbreviation** |
| --- | --- |
| Elevation | Elevation |
| pH | pH |
| Secchi depth | SD |
| Water-body temperature | WT |
| Conductivity | Cond |
| Total dissolved solids | TDS |
| Salinity | Sal |
| Dissolved oxygen | DO |
| Total nitrogen | TN |
| Total phosphorus | TP |
| Chemical oxygen demand | COD |
| Chlorophyll-a | *Chl*-a |
| Arsenic | As |
| Cadmium | Cd |
| Lead | Pb |
| Copper | Cu |
| Zinc | Zn |
| Selenium | Se |
| Distance to Road | DTR |
| Distance to Village | DTV |
| Number of Human in Village | NHV |
| Slope | Slope |
| Bio1 (Annual mean temperature) | AMT |
| Bio2 (Mean diurnal range) | MDR |
| Bio3 (Isothermality) | Isothermality |
| Bio4 (Temperature seasonality) | TS |
| Bio5 (Maximum temperature of warmest month) | MaxT WM |
| Bio6 (Minimum temperature of coldest month) | MinT CM |
| Bio7 (Temperature annual range) | TAR |
| Bio8 (Mean temperature of wettest quarter) | MTWeQ |
| Bio9 (Mean temperature of driest quarter) | MTDeQ |
| Bio10 (Mean temperature of warmest quarter) | MTWaQ |
| Bio11 (Mean temperature of coldest quarter) | MTCoQ |
| Bio12 (Annual precipitation) | AP |
| Bio13 (Precipitation of wettest month) | PWM |
| Bio14 (Precipitation of driest month) | PDM |
| Bio15 (Precipitation seasonality) | PS |
| Bio16 (Precipitation of wettest quarter) | PWeQ |
| Bio17 (Precipitation of driest quarter) | PDQ |
| Bio18 (Precipitation of warmest quarter) | PWaQ |
| Bio19 (Precipitation of coldest quarter) | PCQ |

**Table S2. Polymorphic bands obtained using eight AFLP primer combinations**

| **Primer combinations** | **Sequence (5’- 3’)** | **Polymorphic band** |
| --- | --- | --- |
| E-AGC/M-CTT | E: GACTGCGTACCAATTCAGC  M: GATGAGTCCTGAGTAACTT | 54 |
| E-AGC/M-CAC | E: GACTGCGTACCAATTCAGC  M: GATGAGTCCTGAGTAACAC | 64 |
| E-ACA/M-CTC | E: GACTGCGTACCAATTCACA  M: GATGAGTCCTGAGTAACTC | 56 |
| E-ACA/M-CAT | E: GACTGCGTACCAATTCACA  M: GATGAGTCCTGAGTAACAT | 59 |
| E-ACT/M-CTT | E: GACTGCGTACCAATTCACT  M: GATGAGTCCTGAGTAACTT | 62 |
| E-ACT/M-CGA | E: GACTGCGTACCAATTCACT  M: GATGAGTCCTGAGTAACGA | 59 |
| E-ACT/M-CAA | E: GACTGCGTACCAATTCACT  M: GATGAGTCCTGAGTAACAA | 58 |
| E-ACT/M-CAT | E: GACTGCGTACCAATTCACT  M: GATGAGTCCTGAGTAACAT | 64 |

**Table S3. Polymorphic bands obtained using five MSAP primer combinations**

| **Primer combinations** | **Sequence (5’- 3’)** | **Polymorphic band** |
| --- | --- | --- |
| E-ACT/M-TTA | E: GACTGCGTACCAATTCACT  M: GATGAGTCTAGAACGGTTA | 66 |
| E-AGC/M-TTA | E: GACTGCGTACCAATTCAGC  M: GATGAGTCTAGAACGGTTA | 77 |
| E-AGC/M-TTG | E: GACTGCGTACCAATTCAGC  M: GATGAGTCTAGAACGGTTG | 83 |
| E-ACA/M-TCT | E: GACTGCGTACCAATTCACA  M: GATGAGTCTAGAACGGTCT | 77 |
| E-ACA/M-TTA | E: GACTGCGTACCAATTCACA  M: GATGAGTCTAGAACGGTTA | 89 |

**Table S4.** **Portion of AFLP and MSAP private bands in 9 *V. natans* populations**

|  | **AFLP** | |  | **MSAP** | |
| --- | --- | --- | --- | --- | --- |
| **Species** | **private bands** | **private rate** |  | **private bands** | **private rate** |
| V1 | 64 | 0.16 |  | 49 | 0.19 |
| V2 | 46 | 0.11 |  | 41 | 0.15 |
| V3 | 67 | 0.17 |  | 56 | 0.23 |
| V4 | 70 | 0.18 |  | 52 | 0.21 |
| V5 | 35 | 0.08 |  | 26 | 0.09 |
| V6 | 43 | 0.10 |  | 31 | 0.11 |
| V7 | 54 | 0.13 |  | 43 | 0.16 |
| V8 | 49 | 0.13 |  | 26 | 0.09 |
| V9 | 92 | 0.20 |  | 45 | 0.17 |

**Table S5. Genetic parameters calculated in 9 *V. natans***

| **Species** | **Ia** | **lambda** | **rbarD** |
| --- | --- | --- | --- |
| V1 | 1.87 | 0.875 | 0.005 |
| V2 | 7.393 | 0.952 | 0.018 |
| V3 | 2.156 | 0.889 | 0.005 |
| V4 | 7.298 | 0.833 | 0.018 |
| V5 | 3.325 | 0.857 | 0.009 |
| V6 | 1.582 | 0.900 | 0.004 |
| V7 | 1.400 | 0.909 | 0.003 |
| V8 | 7.427 | 0.958 | 0.018 |
| V9 | 4.372 | 0.923 | 0.012 |

Abbreviations: Ia, index of association for each population factor (Agapow and Burt, 2001), lambda, Simpson’s index (Simpson, 1949); rbarD, standardized index of association for each population factor (Brown et al., 1980; Smith et al., 1993)

**Table S6.** **Rate of methylation types in 9 *V. natans* populations**

| **Species** | **NMSL** | **HMSL** | **INCML** | **FML** |
| --- | --- | --- | --- | --- |
| V1 | 0.042 | 0.092 | 0.171 | 0.696 |
| V2 | 0.032 | 0.139 | 0.109 | 0.720 |
| V3 | 0.042 | 0.089 | 0.155 | 0.715 |
| V4 | 0.039 | 0.098 | 0.147 | 0.717 |
| V5 | 0.027 | 0.097 | 0.151 | 0.725 |
| V6 | 0.034 | 0.123 | 0.121 | 0.722 |
| V7 | 0.029 | 0.067 | 0.085 | 0.820 |
| V8 | 0.055 | 0.084 | 0.080 | 0.781 |
| V9 | 0.131 | 0.138 | 0.054 | 0.677 |

Abbreviations: NMSL, non methylation; HMSL, hemi-methylated proportion; INCML, inner cytosine methylation; FML, full methylated.

**Table S7. Non-hierarchical and hierarchical analysis of molecular variance (AMOVA) of AFLP variation based on groupings of *V. natans* in Liangzi Lake**

| **Source of variation** | **Df** | **Sum of squares** | **Variance components** | **% variance** | **ø-statistic^a^** |
| --- | --- | --- | --- | --- | --- |
| Among populations | 8 | 1751.192 | 11.8 | 13.0 | ø_ST_ = 0.130 |
| Within populations | 100 | 8032.532 | 80.3 | 87.0 | NC |
|  |  |  |  |  |  |
| Two genetic groups |  |  |  |  |  |
| Among groups | 1 | 549.386 | 6.53 | 6.9 | ø_CT_ = 0.069 |
| Among populations within groups | 7 | 1201.806 | 8.02 | 8.5 | ø_SC_ = 0.091 |
| Within populations | 100 | 8032.533 | 80.33 | 84.7 | ø_ST_ = 0.153 |
| Total | 108 | 9783.725 | 94.88 |  |  |

Abbreviations: AMOVA, analysis of molecular variance; Df, degrees of freedom; NC, not computed.

^a^All variance components were significant (*P* < 0.001) based on 999 permutations.

**Table S8. Five types of comparing values in genets and ramets methylated patterns**

| **Types** | **Genet1 with ramets1** | **Genet2 with ramets21** | **Genet21 with ramets22** |
| --- | --- | --- | --- |
| aHash | 0.98 | 0.85 | 0.89 |
| dHash | 0.76 | 0.82 | 0.86 |
| pHash | 0.89 | 0.88 | 0.88 |
| Hist with split | 0.57 | 0.49 | 0.56 |
| Single Hist | 0.65 | 0.44 | 0.49 |

**Table S9.** The results show the top 10 matches of environmental variables with genetic variation of *V. natans* populations.

| **Rank** | ***R*** | **Environmental variables** | ***P*** |
| --- | --- | --- | --- |
| 1 | 0.933 | pH, WT, DO | 0.05 |
| 2 | 0.903 | pH, WT, DO, MTCoQ | 0.05 |
| 3 | 0.890 | pH, WT, DO, TP | 0.05 |
| 4 | 0.883 | pH, WT, DO, *Chl*-a | 0.05 |
| 5 | 0.879 | pH, WT, DO, AP | 0.05 |
| 6 | 0.876 | pH, DO, *Chl*-a | 0.05 |
| 7 | 0.871 | pH, WT | 0.05 |
| 8 | 0.859 | pH, DO, TP, *Chl*-a | 0.05 |
| 9 | 0.847 | pH, DO | 0.05 |
| 10 | 0.844 | pH, DO, *Chl*-a, PDQ | 0.05 |

**Table S10.** The results show the top 10 matches of environmental variables with epigenetic variation of *V. natans* populations.

| **Rank** | ***R*** | **Environmental variables** | ***P*** |
| --- | --- | --- | --- |
| 1 | 0.862 | pH, WT, DO, TP | 0.05 |
| 2 | 0.860 | pH, WT, DO, TP, NHV | 0.05 |
| 3 | 0.852 | pH, WT, DO, TP, DTV | 0.05 |
| 4 | 0.849 | pH, WT, DO | 0.05 |
| 5 | 0.840 | pH, WT, TP | 0.05 |
| 6 | 0.838 | pH, DO, TP, Zn, NHV | 0.05 |
| 7 | 0.834 | pH, WT, DO, Se | 0.05 |
| 8 | 0.834 | pH, DO, TP, Zn, Isothermality | 0.05 |
| 9 | 0.830 | pH, WT, TP, Zn | 0.05 |
| 10 | 0.827 | pH, WT | 0.05 |

**Figure S1**. Minimum spanning networks of the *V. natans* based on Nei’s genetic distance.

**Figure S2.** Results of Bayesian analysis for each samples of *V. natans* from Liangzi Lake. (a-c) Q-plots of *V. natans* genetic clusters assigned by STRUCTURE for *K* = 2-4. In the bar plot each column represents a different individual with the colors showing the probability membership coefficient of that individual for each genetic cluster. Populations are separated by black bars and identified at bottom. (d) The estimation of an ad hoc quantity (∆*K*) from calculated *K* and LnP (D).

**Figure S3.** The top 19 environmental variables associated with genetic and epigenetic variation. (a) RF model detected the association between genetic/epigenetic variation and 41 environmental variables. (b) MIC analysis detected the importance of environmental variables associated to genetic and epigenetic variations in *V. natan* populations.

**Figure S4.** Genetic variants outiers associated with the environmental variables. (a) Quantile-Quantile plot for -Log10 *P*-values from association tests. (b) Manhattan plot for genetic variants associated with the environmental gradient. The upper straight lines represent significance thresholds (FDR correction, adjusted *P* = 0.01). Selected candidate locis are labeled in the plot at their respective probe positions.

**Figure S5.** Epigenetic variants outiers associated with the environmental variables. (a) Quantile-Quantile plot for -Log10 *P*-values from association tests. (b) Manhattan plot for epigenetic variants associated with Water-body temperature (WT) (blue point), Total dissolved solids (TDS); Salnity (Sal) (violet point), and *Chl*-a (darkgreen point). The gray dashed horizontal lines represent significance thresholds (Bonferroni correction, adjusted *P* = 0.05). The upper straight lines represent significance thresholds (FDR correction, adjusted *P* = 0.01). Selected candidate locis are labeled in the plot at their respective probe positions.

**Figure S6**. Differences of methylated types between genets and ramets among *V. natans* individuals. (a) Specific methylation levels of 22 *V. natans* individuals between genets and ramet. (b) 8 individuals of methylation variances among genets, ramets1, and ramets2.

**Supporting information references:**

Vos, P., Hogers, R., Bleeker, M., Reijans, M., Lee, T. v. d., Hornes, M., . . . Kuiper, M. (1995). AFLP: a new technique for DNA fingerprinting. *Nucleic acids research, 23*(21), 4407-4414.

Baurens, F.-C., Causse, S., & Legavre, T. (2008). Methylation-sensitive amplification polymorphism (MSAP) protocol to assess CpG and CpNpG methylation in the banana genome. *Fruits, 63*(2), 117-123.

Brown, A., Feldman, M., Nevo, E., 1980. Multilocus structure of natural populations of Hordeum spontaneum. Genetics 96, 523-536.

Simpson, E.H., 1949. Measurement of diversity. Nature 163, 688-688.

Smith, J.M., Smith, N.H., O'Rourke, M., Spratt, B.G., 1993. How clonal are bacteria? Proceedings of the National Academy of Sciences 90, 4384-4388.
